# Enhanced Cancer Starvation Therapy Based on Glucose Oxidase/3-Methyladenine-Loaded Dendritic Mesoporous OrganoSilicon Nanoparticles

**DOI:** 10.3390/biom11091363

**Published:** 2021-09-14

**Authors:** Fan Wu, Yang Liu, Hui Cheng, Yun Meng, Jieyun Shi, Yang Chen, Yelin Wu

**Affiliations:** 1 Tongji University Cancer Center, Shanghai Tenth People’s Hospital, Tongji University School of Medicine, Shanghai 200072, China; 18844191898@163.com (F.W.); 18356070780@163.com (Y.M.); 15800870206@163.com (J.S.); fc9091@163.com (Y.C.); 2 Shanghai Key Laboratory of Green Chemistry and Chemical Processes, School of Chemistry and Molecular Engineering, East China Normal University, Shanghai 200062, China; ly92_pharmacy@163.com (Y.L.); haotianchenghui@163.com (H.C.)

**Keywords:** mesoporous nanomaterials, organosilicon, anti-cell adaptation, autophagy inhibition, starvation therapy

## Abstract

Cell autophagy is a well-known phenomenon in cancer, which limits the efficacy of cancer therapy, especially cancer starvation therapy. Glucose oxidase (GOx), which is considered as an attractive starvation reagent for cancer therapy, can effectively catalyze the conversion of glucose into gluconic acid and hydrogen peroxide (H_2_O_2_) in the presence of O_2_. However, tumor cells adapt to survive by inducing autophagy, limiting the therapy effect. Therefore, anti-cell adaptation via autophagy inhibition could be used as a troubleshooting method to enhance tumor starvation therapy. Herein, we introduce an anti-cell adaptation strategy based on dendritic mesoporous organosilica nanoparticles (DMONs) loaded with GOx and 3-methyladenine (3-MA) (an autophagy inhibition agent) to yield DMON@GOx/3-MA. This formulation can inhibit cell adaptative autophagy after starvation therapy. Our in vitro and in vivo results demonstrate that autophagy inhibition enhances the efficacy of starvation therapy, leading to tumor growth suppression. This anti-cell adaptation strategy will provide a new way to enhance the efficacy of starvation cancer therapy.

## 1. Introduction

Deriving from an ancient concept of anti-angiogenesis, cancer starvation therapy implemented via ensuring an insufficient supply of nutrition and oxygen by obstructing blow vessels, has been proposed as a new antitumor approach [1,2]. With the advancements of nanotechnology, our research group first synthesized Mg_2_Si nanoparticles for direct oxygen consumption in the tumor. These Mg_2_Si nanoparticles produced SiO_2_, which aggregates in tumor capillaries, causing extensive devascularization, and thus achieving significant tumor growth inhibition [3]. Subsequently, nanomaterials with deoxygenation ability were progressively developed for this starvation therapy [4,5]. Notably, the glucose that cells take up from the blood is the major nutritive source of energy for cell growth and cell metabolism via aerobic glycolysis [6]. In contrast to normal cells, more glucose is needed for cancer cells to meet their energy requirement for tumor growth and metastasis based on the Warburg effect [7,8]. Therefore, glucose depletion strategies have been extensively used in cancer starvation therapy. As widely known, glucose oxidase (GOx) can catalyze the oxidation of glucose to gluconic acid and hydrogen peroxide (H_2_O_2_) in the presence of O_2_, leading to the simultaneous consumption of oxygen and glucose, and consequently starvation [9,10,11]. However, cells under this metabolizing pressure of starvation and acidosis will activate autophagy, one of the cellular adaptative mechanisms, to prolong their survival and further weaken the therapeutic effect of starvation [12]. Therefore, the therapeutic effect of GOx alone is limited, and the current trend in GOx-based cancer therapy is to combine with other therapies such as chemotherapy [13], photodynamic therapy (PDT) [14], photothermal therapy (PTT) [15], and chemodynamic therapy (CDT) [16].

Autophagy is a ubiquitous process that induces the breakdown of abnormal cellular constituents within lysosome in response to a variety of stress conditions, including DNA damage, chemotherapy, protein folding errors, microbial infection, organelle injury, radiotherapy, or starvation [17,18,19], and then allows cells to adapt to environmental and/or developmental changes [20]. Therefore, inhibiting autophagy will undoubtedly enhance the treatment effect of tumor starvation therapy [21,22,23,24]. Typically, 3-methyladenine (3-MA) has been proven to be an effective autophagy inhibitor via inhibiting the class III PI3K (Vps34)/Beclin-1 complex [25,26,27]. To achieve simultaneous enrichment of GOx and 3-MA in a tumor, an intrinsically non-toxic drug delivery system should be used. Dendritic mesoporous organosilicon nanoparticles (DMON) are constructed as nanocarriers with significant advantages over the conventional inorganic silica, e.g., enhanced drug loading capacity and effective chemotherapeutics leakage prevention [28,29,30,31,32]. DMONs have been used to load GOx and ultrasmall Fe_3_O_4_ for fabrication of the sequential nanocatalyst, which exhibits excellent catalytic performances for efficient tumor therapy [16]. Thus, incorporating GOx and 3-MA into DMONs is highly promising for tumor-specific therapeutics both in vitro and in vivo.

Motivated by these facts, we constructed DMON nanoparticles for co-delivery GOx and 3-MA, creating the nanoparticles DMON@GOx/3-MA, which releases its 3-MA to enhance GOx-induced cancer starvation. As shown in Equation (1), GOx catalyzed the oxidation process of glucose to gluconic acid and H_2_O_2_ through the following reaction:

(1) $$\mathrm{Glucose}\;+{\;O}_{2}+{\;H}_{2}0\;\overset{\mathrm{GOx}}{\to }\mathrm{Gluconic}\;\mathrm{acid}+{\;H}_{2}O_{2}$$

**Figure 1:**
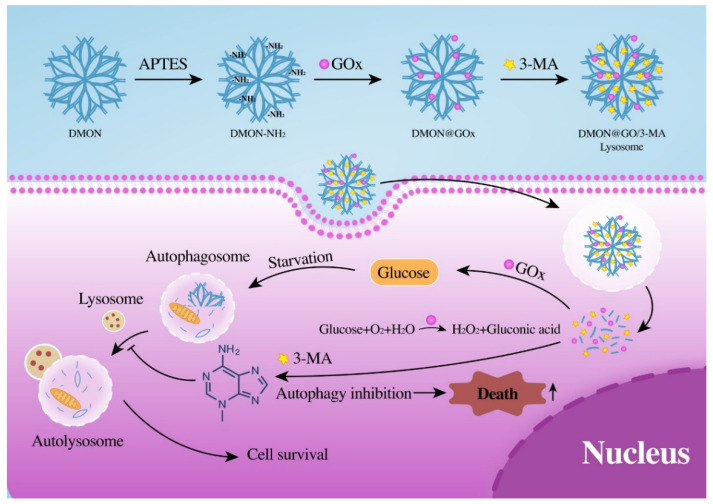
Schematic representation of the functional pattern of DMON@GOx/3-MA. Autophagy inhibitor 3-MA released from DMON@GOx/3-MA enhances GOx-induced starvation therapy in tumor cells.

The change in the intracellular environment caused by the lowered levels of oxygen and glucose activates autophagy to protect cells from energy starvation. However, this process is efficiently inhibited by 3-MA, sensitizing GOx-induced tumor starvation therapy (Figure 1). Overall, the study provides an effective anti-cell adaptation strategy to inhibit autophagy, enhancing starvation therapy by co-loading GOx and 3-MA. This anti-cell adaptation strategy will provide a new method to enhance the efficacy of starvation cancer therapy.

## 2. Materials and Methods

### 2.1. Materials

Cetyltrimethylammonium bromide (CTAB), tetraethyl orthosilicate (TEOS), 1,2-bis(triethoxysilyl)ethane (BTEE), triethanolamine (TEA), glucose oxidase (GOx), 3-methyladenine (3-MA) and sodium salicylate (NaSal) were purchased from Sigma-Aldrich (Shanghai, China). 1-Ethyl-3(3-dimethylpropylamine) carbodiimide (EDCI) and N-hydroxysulfosuccinimide (NHS) were purchased from Macklin (Shanghai, China). Ethanol and methanol were purchased from Sinopharm Chemical Reagent Co., Ltd. (Shanghai, China). All chemicals were used as received without further purification.

### 2.2. Synthesis of Dendritic Mesoporous Organosilicon Nanoparticles (DMONs)

In a typical synthesis of DMONs, TEA (0.068 g) was added to water (25 mL) and stirred at 80 °C in an oil bath for 0.5 h. Then, CTAB (380 mg) and NaSal (126 mg) were added to the above solution with stirring for 5 h. Subsequently, a mixture of BTEE (1.6 mL) and TEOS (2 mL) was added to the abovementioned solution with vigorous stirring for 12 h. Then, the products were collected by high-speed centrifugation (11,544× *g*, 10 min), washed three times with 20 mL ethanol to remove the residual reactants, and finally redispersed in ultrapure ethanol. Next, DMON nanoparticles were then functionalized with amino (-NH_2_) groups. First, the DMON nanoparticles were dispersed in ethanol (50 mL, 10 mg/mL) and placed in an oil bath (78 °C) with magnetic stirring. Then 3-aminopropyltriethoxysilane (APTES) (50 μL) was added into the above solution and kept stirring for another 12 h. Finally, the amino decorated DMONs (DMON-NH_2_) were collected by centrifugation (11,544× *g*, 10 min), washed three times with ethanol (20 mL) and deionized water (20 mL) before being redispersed in deionized water (50 mL) for further use.

### 2.3. Synthesis of DMON@GOx

For the synthesis of DMON@GOx nanoparticles, GOx powder (2 mg) was added to DMON-NH_2_ (10 mL, 1 mg/mL) water solution with vigorous magnetic stirring. Then, EDCI (2.1 mg, 2 mmol) and NHS (1.5 mg, 2 mmol) were added to the resulting solution. After 12 h of reaction, the GOx decorated DMON-NH_2_ (DMON@GOx) nanoparticles were collected by centrifugation (11,544× *g*, 10 min) and washed three times with ethanol (20 mL) and deionized water (50 mL) before being redispersed in deionized water for further use.

### 2.4. Synthesis of DMON@GOx/3-MA

For the synthesis of DMON@GOx nanoparticles, 3-MA (2 mg) was added into DMON/GOx (10 mL, 1 mg/mL) water solution with vigorous magnetic stirring. After 12 h of reaction, the 3-MA was loaded onto the dendritic pores of DMON@GOx nanoparticles. Then, DMON@GOx/3-MA nanoparticles were collected by centrifugation (11,544× *g*, 10 min).

### 2.5. Synthetic of FITC-Labeled DMON@GOx/3-MA

For the synthesis of fluorescein isothiocyanate (FITC)-labeled DMON@GOx/3-MA nanoparticles, FITC (1 mg) was added into DMON/GOx/3-MA (10 mL, 1 mg/mL) water solution under vigorous magnetic stirring. After 8 h, FITC was loaded on the dendritic pores of DMON@GOx/3-MA nanoparticles. Then, FITC-labeled DMON@GOx/3-MA nanoparticles were collected by centrifugation (11,544× *g*, 10 min) and washed three times with deionized water (20 mL) to remove the unloaded FITC.

### 2.6. Characterizations

X-ray diffraction (XRD) patterns were analyzed on an Ultima IV X-ray diffractometer (Rigaku, Tokyo, Japan) utilizing Cu Kα (λ = 1.5405 Å) radiation along with scan range from 10° to 80°. Transmission electron microscopy (TEM) images were captured on a JEOL JEM-2100 microscope operated at 200 kV (JEOL Ltd., Tokyo, Japan). All the TEM samples were obtained by dropping dilute nanomaterials onto carbon coated copper grids. The particle size and zeta potential analyzers were measured on Microtrac Nanotrac Wave II Dynamic light scattering (Microtrac Inc., Philadelphia, PA, USA). Ultraviolet Visible (UV-VIS) spectra were recorded on a UV-3600 Plus spectrophotometer (Shimadzu, Tokyo, Japan). Fourier transform infrared (FTIR) spectra were recorded on a TENSOR II FT-IR spectrophotometer (Bruker Corporation, Karlsruhe, Germany) using KBr pellets. CLSM images were obtained on a Nikon A1Rt-980 confocal microscope (Nikon Corporation, Tokyo, Japan).

### 2.7. Catalytic Ability Measurement

To measure the catalytic ability of DMON@GOx nanoparticles, different concentrations of glucose solutions (0, 12.5, 25, 50, 100, and 200 μg/mL) were added to DMON@GOx solution (GOx = 0.5 mg/mL) in an open 96-well plate. After 2 h of sufficient catalytic reaction, 50 μL of titanous sulfate reagent (20 mM) was added to each well. Then the absorbance of each well at 415 nm was recorded using a Spark microplate reader (Tecan, Shanghai, China).

### 2.8. 3-MA Release Performance

In order to release the performance of 3-MA in simulated body fluid (SBF) solution, one milliliter of DMON@GOx/3-MA (10 mg/mL) was added to the dialysis bag (cut-off weight = 12,000–14,000 Da) and then transfer to a beaker with 20 mL of SBF buffer (pH = 7.4 or 5.4). At different time points, one milliliter of SBF solution was collected and measured by high phase liquid chromatography (HPLC). HPLC parameter settings—Mobile phase: ammonium acetate (30 mM): acetonitrile = 95:5 (by volume); Determine wavelength = 240 nm. Chromatographic column: C-18 reverse-phase column.

### 2.9. Cell Culture

4T1 cells were obtained from the Shanghai Institute of Cells, Chinese Academy of Sciences. The 4T1 cells were cultured in Dulbecco’s modified Eagle medium (DMEM) (Gibco, Shanghai, China) with 10% heat-inactivated fetal bovine serum (FBS), streptomycin (100 mg/mL) and penicillin (100 U/mL). The cells were incubated in standard incubators at 37 °C, in a 5% CO_2_ atmosphere.

### 2.10. Cell Cytotoxicity Assessment

The cytotoxicity of the DMON@GOx, DMON@GOx/3-MA and glucose on the 4T1 cells were evaluated using the standard methyl thiazolyl tetrazolium (MTT) method, and each data point was described using the mean and standard deviation of 6 replicates. The specific experimental procedure was as follows: The 4T1 cells were seeded into 96-well plates at a density of 1 × 10^4^ per well, then treated with different concentrations of DMON@GOx, DMON@GOx/3-MA, or glucose for 24 h. Next, the culture medium was discarded, and 100 μL of MTT medium solution (0.8 mg/mL) was added to each well. Each plate was then incubated at 37 °C for 4 h. After incubation, the MTT medium solution was aspirated, and 100 μL of DMSO solution was added to each well. The OD_490_ value of each well was then measured on a Bio-Tel ELx800 microplate reader using a detection wavelength of 490 nm. The cell survival rate was calculated using the following formula: Cell viability (%) = (OD_490_ value of sample/blank OD_490_ value) × 100%.

### 2.11. Western Blot Assay

4T1 cells plated into 6-well plates were treated with DMON@GOx and DMON@GOx/3-MA for 24 h. The cells were lysed with 100 μL of RIPA lysis buffer (150 mM NaCl, 1% IGEPAL CA-630, 0.5% SDS, 50 mM Tris-HCL, pH 8.0) supplemented with protease inhibitor (PMSF 0.5 mM). The equal amounts of protein added with 2× Laemmli loading buffer (4% SDS, 10% 2-mercaptoethanol, 20% glycerol, 0.004% bromophenol blue, and 0.125 M Tris-HCl) were denatured by boiling the sample at 100 °C for 10 min. After that, the proteins were separated on 12% SDS-PAGE and transferred to polyvinylidene fluoride (PVDF) membranes (Millipore, Bedford, MA, USA). After blocking with 5% nonfat milk, the PVDF membrane was incubated with a primary antibody, such as anti-P62, anti-LC3B, anti-caspase3, Actin and GAPDH (Cell Signaling Technology, Boston, MA, USA) at 4 °C overnight. Then the membrane was incubated with a related secondary antibody (Alexa Fluor 680 Goat anti-Rabbit IgG (H + L) and Alexa Fluor 690 Goat anti-Mouse IgG (H + L), Jackson, West Grove, PA, USA) for 1 h at room temperature, and finally scanned using an Odyssey infrared imager (LI-COR Biosciences, Corston, UK).

### 2.12. Bio-TEM Analysis of Cancer Cells

First, 4T1 cells (10^6^/dish) treated with 400 μg/mL of DMON@GOx/3-MA were placed at 37 °C incubator for 24 h. The cells were then washed twice with PBS buffer and digested with 0.25% trypsin. The cells were collected by centrifugation (300× *g*, 3 min) and fixed with 1 mL glutaraldehyde at room temperature. Then, washed with 1 mL PBS buffer, the cells were dehydrated with a gradient of ethanol (30%, 50%, 75%, 80%, 95%, 100%) and washed with 1 mL propylene oxide. The cells were embedded in epoxy resin EPOM812 and polymerized at 37 °C for 12 h, 45 °C for 12 h and 60 °C for 48 h. The epoxy resin was cut into 70 nm sections, poststained with 4% uranyl acetate and 2.5% glutaraldehyde for 10 min and imaged at 100 kV on a transmission electron microscope (TEM).

### 2.13. Apoptosis Assay

4T1 cells seeded in 6-well plates (3 × 10^4^/well) were treated with PBS, DMON@GOx, and DMON@GOx/3-MA for 24 h. The suspended and adherent cells in each well were collected by centrifugation, washed twice with PBS, and stained with 2 μL of fluorescein isothiocyanate (FITC)-labeled annexin V (Annexin V-FITC) and 2 μL of propidium iodide at room temperature for 30 min in the dark. The apoptotic cells were detected by flow cytometry and analyzed using the FlowJo software.

### 2.14. Immunofluorescence

4T1 cells seeded in confocal dishes were treated with PBS, DMON@GOx, DMON@GOx/3-MA for 24 h. Cells were fixed with 4% paraformaldehyde for 30 min and permeabilized with 0.2% Triton-100 for 15 min. Then blocked with PBS with 1% BSA at room temperature for 1 h. The cells were incubated with primary antibody, P62 (1 μg/mL) and LC3B (1 μg/mL) at 4 °C overnight. Cells were incubated with secondary antibody (1:500) at room temperature for 1 h. Then incubated with phalloidins (1:400) at room temperature for 1 h. Finally, all samples were incubated with DAPI for 10 min and observed by CLSM.

### 2.15. Immunofluorescence of tfLC3

4T1 cells were seeded in confocal dishes, then infected with Ad-virus expressing mRFP-GFP-LC3 (tfLC3) (Hanbio, Shanghai, China) 2 μL/dish, refresh the medium after 6 h. After infected 24 h, treated with PBS, DMON@GOx, DMON@GOx/3-MA for 24 h. The cells were examined under CLSM.

### 2.16. Animals and Tumor Model

All animal experiments were performed according to the Principles of Laboratory Animal Care (China) and the Guidelines of the Animal Investigation Committee. Animal testing procedures followed the guidelines of the Animal Protection and Use Committee.

### 2.17. Therapeutic Assessment In Vivo

4T1 cells (2 × 10^6^ cell/site) were injected subcutaneously into the female Balb/c nude mice (~20 g). In vivo therapy experiments were performed when the tumor reached 6 mm in average diameter (7 days after implant). The mice were divided into five groups. The first group of mice received PBS, as the control group; the second group was intravenously injected with DMON@GOx once a day for day 1–3 (10 mg/mL, in 150 μL PBS), as DMON@Gox (i.v.) group; the third group was intravenously injected with DMON@GOx/3-MA once a day for day 1–3 (10 mg/mL, in 150 μL PBS), as DMON@GOx/3-MA (i.v.) group; the fourth group was intratumorally injected with DMON@GOx at day 1 (10 mg/mL, in 150 μL PBS), as DMON@GOx (i.t.) group; the fifth group was intratumorally injected with DMON@GOx/3-MA at day 1 (10mg/mL, in 150 μL PBS), as DMON@GOx/3-MA (i.t.) group. After the corresponding treatments, the volume of each tumor was measured every other day and calculated by the following equation: V = L × W^2^/2. Furthermore, the tumors were sectioned into slices for TUNEL staining.

### 2.18. In Vivo Toxicity Assessment of DMON@GOx/3-MA

Healthy female Kunming mice (~20 g) were purchased from Beijing Vital River Laboratory Animal Technology Co., Ltd. (Beijing, China). These mice were intravenously injected with DMON@GOx/3-MA at a calculated dose of 75 mg/kg. The mice were sacrificed at day 0, 3 and day 30. The major organs (heart, liver, spleen, lung, and kidney) were dissected, fixed in a 10% formalin solution and stained with hematoxylin and eosin (H&E) for histological analysis. Alanine aspartate aminotransferase (AST), nephric blood urea nitrogen (BUN), aminotransferase (ALT), alkaline phosphatase (AKP) and creatinine (CREA) indicators, together with the complete blood panel parameters including red blood cells (RBC), white blood cells (WBC), red blood cell distribution width-standard deviation (RDW-SD), hemoglobin (HGB), mean corpuscular volume (MCV), mean corpuscular hemoglobin concentration (MCHC), mean corpuscular hemoglobin (MCH), hematocrit (HCT) and lymphocyte (LYM) of all groups by using authoritative standard biochemistry test.

## 3. Results

### 3.1. Synthesis and Characterization of DMON@GOx/3-MA

**Figure 2:**
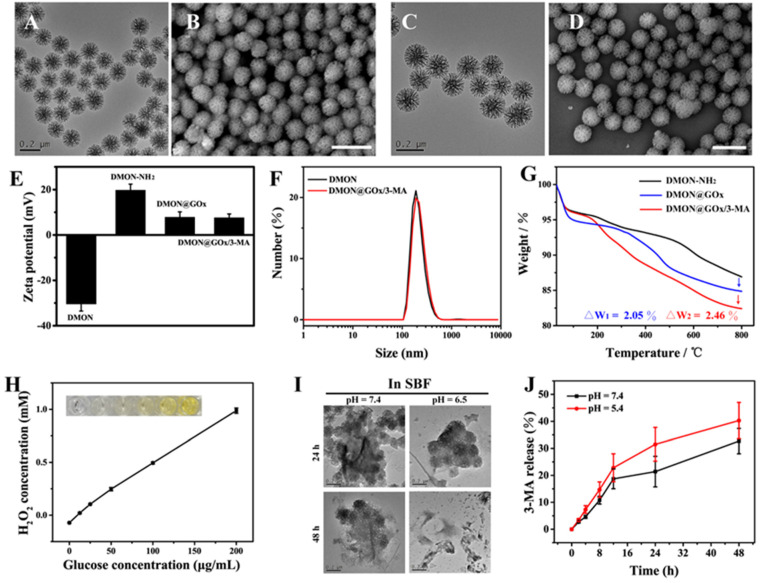
Characterization of DMON@GOx/3-MA nanoparticles. Transmission electron microscopy (TEM) (**A**) and scanning electron microscopy (SEM) (**B**) images of DMON nanoparticles (scale bar = 200 nm). TEM (**C**) and SEM (**D**) images of DMON@GOx/3-MA nanoparticles (scale bar = 200 nm). (**E**) Zeta potential of different nanoparticles during the synthetic process (n = 3, mean ± s.d.). (**F**) Dynamic light scattering (DLS) data of DMON and DMON@GOx/3-MA nanoparticles in deionized water. (**G**) Thermogravimetric (TG) curves of DMON-NH_2_, DMON@GOx, and DMON@GOx/3-MA nanoparticles. The arrows indicate the decrease of weight. (**H**) The generated H_2_O_2_ concentration after co-incubation DMON/GOx nanoparticles at different concentrations of glucose for 2 h (n = 3, mean ± s.d.). (**I**) TEM images of DMON@GOx/3-MA nanoparticles after dispersed in different simulated body fluid (SBF) buffer (pH = 7.4 and 5.4) for 24 and 48 h (Scale bar = 200 nm). (**J**) Release kinetics of 3-MA from DMON@GOx/3-MA (n = 3, mean ± s.d.).

The biodegradable DMONs were synthesized via the hydrolysis of tetraethyl orthosilicate (TEOS) and 1,2-bis(triethoxysilyl)ethane (BTEE) with the assistance of cetyltrimethylammonium bromide (CTAB) and sodium salicylate (NaSal). As the transmission electron microscopy (TEM) and scanning electron microscopy (SEM) images in Figure 2A,B show, monodispersed dendritic nanoparticles were prepared successfully. The X-ray diffraction (XRD) data of DMON also matched well with the pattern of simulated SiO_2_ (PDF: 39-1425, Figure S1).

After that, the surface of DMON was functionalized by -NH_2_ groups for the further modification of GOx, as shown in Figure 2E, the zeta potential of DMON-NH_2_ turned negative (−30.4 mV) to positive (+19.7 mV) after the -NH_2_ functionalization. Then the GOx was loaded on DMON by the condensation reaction of NH_2_- (in DMON-NH_2_) and -COOH (in GOx). In the end, the 3-MA was loaded on the dendritic pores of DMON@GOx and formed the final formulation (DMON@GOx/3-MA). As indicated in Figure 2C,D, the DMON@GOx/3-MA nanoparticles remained well dispersity and the structures did not have obvious change. This result was also confirmed by the dynamic light scattering (DLS) data (Figure 2F), both DMON and DMON@GOx/3-MA nanoparticles showed good uniformity with the hydrated particle size of 190 and 198 nm respectively. In addition, DMON@GOx/3-MA still maintains good dispersibility in a variety of physiological solutions (Figure S2). Besides, the FTIR spectra of GOx, 3-MA, DMON-NH_2_ and DMON@GOx/3-MA were recorded. As we can see from Figure S3, both the characteristic peaks of GOx and 3-MA can be found in the spectrum of DMON@GOx/3-MA, which proved that the GOx and 3-MA were loaded successfully. Further, thermogravimetric (TG) analysis of GOx, 3-MA, DMON-NH_2_, DMON@GOx and DMON@GOx/3-MA were carried out to determine the loading capacities of GOx and 3-MA. By comparing the weight loss curves of different samples (Figure 2G and Figure S4), the loading capacities of GOx = ΔW1/(1−11.14%) = 2.31%, and 3-MA = ΔW2/(1−0.95%) = 2.48%.

In order to verify the catalytic ability of GOx, DMON@GOx was incubated with different concentrations of glucose solution, and the generated H_2_O_2_ was quantified by a classic titanium sulfate colorimetric method according to the H_2_O_2_ standard curve (Figure S5). As shown in Figure 2H, the amount of H_2_O_2_ produced by the GOx catalytic process increased by the increased concentration of glucose, which indicated that the modified GOx on DMON remained catalytic activity.

To evaluate the biodegradation and 3-MA release performance of DMON@GOx/3-MA, simulated body fluid (SBF) media with different pH (7.4 and 5.4) values were used to imitate the in vivo neutral healthy body fluids and mildly acidic tumor microenvironment. As shown in Figure 2I, the morphology of DMON@GOx/3-MA was collapsed both in neutral and acidic SBF media after 24 h incubation, and the structure showed obvious degradation after 48 h, especially in an acidic medium. Further, the release performance of 3-MA in SBF media was also studied. Cumulatively, about 32.7% and 40.3% of 3-MA were released under pH = 7.4 and 5.4 respectively in 48 h (Figure 2J). These results indicated that the as-obtained DMON@GOx/3-MA were biodegradable and possessed the potential to be used in vivo.

### 3.2. In Vitro Bioactivity of DMON@GOx/3-MA

**Figure 3:**
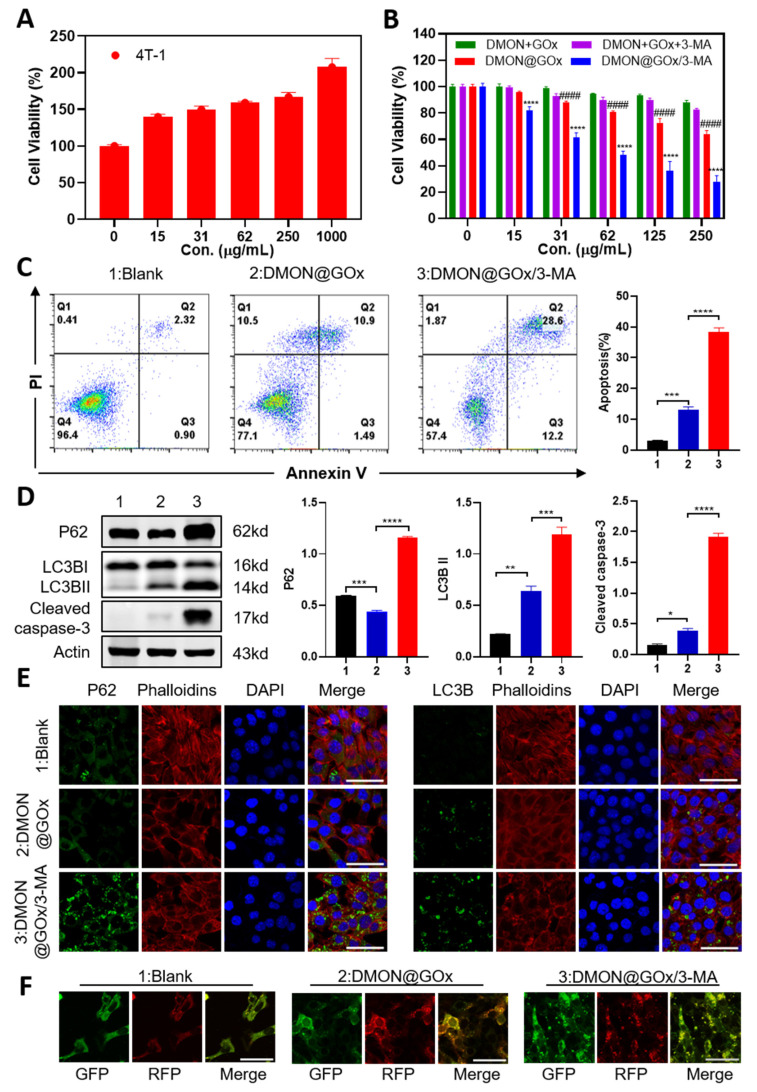
In vitro bioactivity of DMON@GOx/3-MA. (**A**) Cell viability of 4T1 cells treated with different concentrations of glucose for 24 h. (**B**) Cell viability of 4T1 cells treated with different concentrations of DMON@GOx, DMON@GOx/3-MA, DMON + GOx, and DMON + GOx + 3-MA. (* comparision between DMON@GOx and DMON@GOx/3-MA, ^#^ comparision between DMON@GOx and DMON + GOx). (**C**) Cell apoptosis analysis detected by PI and AnnexinV in 4T1 cells treated with 125 μg/mL DMON@GOx and DMON@GOx/3-MA for 24 h. (**D**) Western blot and the grey level analysis of 4T1 cells treated as (**C**). (**E**) Immunofluorescence of P62 and LC3B of 4T1 cells treated as (**C**). (**F**) Immunofluorescence of tfLC3 puncta of 4T1 cells treated as (**C**). Scale bar: 50 μm. * *p* < 0.1, ** *p* < 0.01,*** *p* < 0.001, **** *p* < 0.0001, ^####^ *p* < 0.0001, *p* value was analysed by one-way ANOVA. Data are the means ± s.d.

To determine the biological activity of DMON@GOx/3-MA, the process of endocytosis of DMON@GOx/3-MA was firstly visualized via confocal laser scanning microscopy (CLSM). As shown in Figure S6, FITC-labeled DMON@GOX/3-MA was easily and efficiently uptaken by the 4T1 tumor cells after incubation for 4 h. Secondly, the cytotoxicity function of glucose, 3-MA, DMON + GOx, DMON + GOx + 3-MA, DMON@GOx, and DMON@GOx/3-MA in 4T1 cells were evaluated by a typical methylthiazolyltetrazolium (MTT) assay. As shown in Figure 3A, glucose promoted the cell viability of 4T1 cells, suggesting glucose is required for the proliferation of 4T1 cells. In contrast, consumption of glucose by DMON@GOx/3-MA, DMON@GOx, DMON + GOx + 3-MA, and DMON + GOx groups inhibited the cell proliferation in different degrees, suggesting glucose is important for the survival of 4T1 cells (Figure 3B). As shown in Figure 3B, DMON@GOx exhibited higher cytotoxicity than DMON + GOx groups, indicating nanoparticles have higher cytotoxicity than the mixtures of the individual components. Furthermore, DMON@GOx/3-MA exhibited higher cytotoxicity than DMON@GOx, indicating autophagy inhibition significantly enhanced GOx-induced cytotoxicity. And this cytotoxicity of DMON@GOx/3-MA is independent of 3-MA toxicity, as the concentration of 3-MA from DMON@GOx/3-MA is about 6.51 μg/mL (the loading capacity of 3-MA = ΔW2/(1–0.95%) = 2.48%; 250 × 2.48/95.21 = 6.51 μg/mL), which is a relatively nontoxic concentration (Figure S7).

To further evaluate the activity of DMON@GOx/3-MA, a cell apoptosis assay was performed. Similar to the result of MTT, PI/Annexin V staining showed that DMON@GOx/3-MA triggered 40.80% cell apoptosis in 4T1 cells, which was nearly three times more than DMON@GOx did (cell apoptotic rate 12.39%, Figure 3C). These results were consistent with the expression pattern of cell apoptotic marker cleaved caspase 3 detected by Western blot (Figure 3D). All these results demonstrated that 3-MA enhanced GOx-induced tumor starvation therapy.

To further demonstrate whether 3-MA-enhanced tumor starvation therapy is through cell autophagy, we then detected the autophagy using Western blot, immunofluorescence, and Bio-TEM in 4T1 cells treated with DMON@GOx and DMON@GOx/3-MA. As shown in Figure 3D, DMON@GOx significantly increased LC3BII production and inhibited P62 expression, suggesting DMON@GOx induces autophagy. However, the supplement of 3-MA significantly increased GOx-induced LC3BII production, meanwhile enhanced GOx-inhibited P62 expression. This result was further confirmed by the immunofluorescence, which exhibited increased LC3BII production and P62 expression after DMON@GOx/3-MA administration (Figure 3E), suggesting 3-MA inhibits GOx-induced autophagy via blocking autophagy flux but not the formation of the autophagosome.

To further verify 3MA inhibited autophagy flux, we performed a tandem fluorescent mRFP-GFP-LC3 (tfLC3) assay, a valuable tool for examining autophagosome maturation and autolysosome formation [33,34]. LC3 puncta labeled with both GFP and mRFP represent autophagosomes. when autophagosomes fuse with lysosomes to form autolysosomes, the GFP loses fluorescence due to lysosomal acidic. Those labeled with mRFP alone represent autolysosomes. As shown in Figure 3F, the red and green puncta in the DMON@Gox and DMON@Gox/3-MA groups were more than those in the blank group, suggesting an increase of autophagosomes in these two groups. Treatment with DMON@Gox led to more red puncta than green puncta, demonstrating the formation of autolysosome and the autophagy flux is normal. However, treatment with DMON@Gox/3-MA led to almost equal numbers of red and green puncta, demonstrating the formation of autolysosome is inhibited and autophagy flux is blocked. This result suggested that the addition of 3-MA enhances the cytotoxicity of DMON@GOx through repressing autophagy. We then performed the Bio-TEM experiment to observe the number of autophagosomes. As shown in Figure S8, DMON@GOx/3-MA administration increased the number of autophagosomes, when compared with DMON@GOx. This result demonstrates that 3-MA inhibits DMON@GOx-induced autophagy via blocking autophagy flux, resulting in the accumulation of autophagosomes, finally enhancing GOx-induced tumor starvation therapy.

### 3.3. In Vivo Tumor Growth Inhibition by DMON@GOx/3-MA

**Figure 4:**
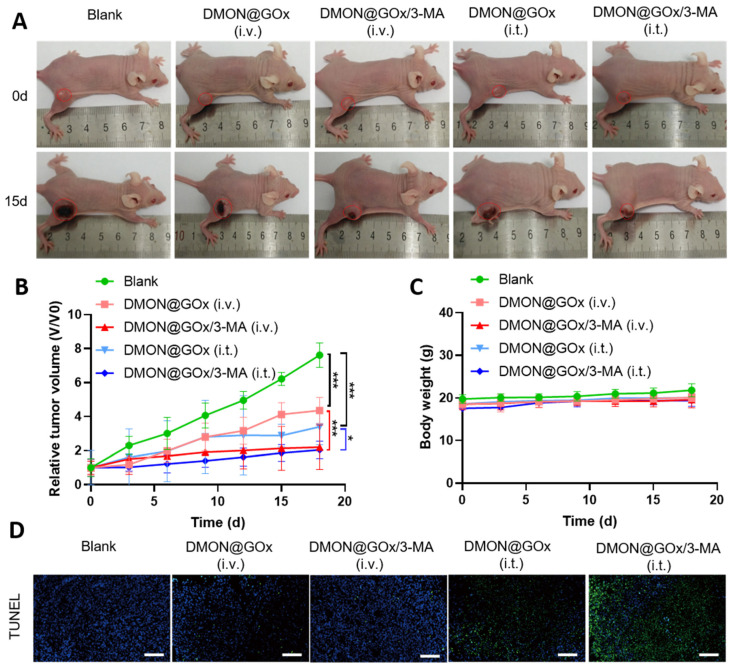
In vivo bioactivity of DMON@GOx/3-MA. (**A**) Digital pictures of tumors from the 4T1 tumor-bearing nude mice i.v. or i.t. injected with DMON@GOx or DMON@GOx/3-MA. (**B**) Tumor growth curves of 4T1 tumor-bearing nude mice treated as in (**A**). (**C**) Body weight of the mice treated as in (**A**). (**D**) TUNEL staining of tumor sections from the 4T1 tumor-bearing mice. Scale bar: 100 μm. * *p* < 0.1, *** *p* < 0.001, *p* value was analysed by two-way ANOVA. Data are the means ± s.d.

Encouraged by these in vitro experiments, we tested the tumor inhibition effect of DMON@GOx/3-MA in 4T1 tumor-xenografted nude mice. 4T1 cells (2 × 10^6^ cell/mouse) were subcutaneously implanted into female Balb/c nude mice (~20 g) and then intravenous injection (i.v.) or intratumoral injection (i.t.) with PBS, DMON@GOx or DMON@GOx/3-MA when the tumor diameter reached about 6 mm. DMON@GOx and DMON@GOx/3-MA significantly inhibited tumor growth in 4T1 cells no matter i.v. or i.t. injection, however, the tumor growth rate in mice treated with DMON@GOx/3-MA (i.t. or i.v.) was much slower than those tumors treated with DMON@GOx (i.t. or i.v.), respectively, demonstrating autophagy inhibition enhanced GOx-induced starvation therapy (Figure 4A,B and Figure S9). In addition, all mice had stable body weights during the treatment period, indicating a negligible biotoxicity (Figure 4C). Consistently, as shown in Figure 4D, TUNEL staining revealed that tumors treated with DMON@GOx/3-MA (i.t. or i.v.) had much more apoptotic cells than tumors treated with DMON@GOx (i.t. or i.v.) further demonstrating autophagy inhibition by 3-MA enhanced GOx-induced tumor starvation therapy.

### 3.4. Low Systematic Toxicity of DMON@GOx/3-MA In Vivo

**Figure 5:**
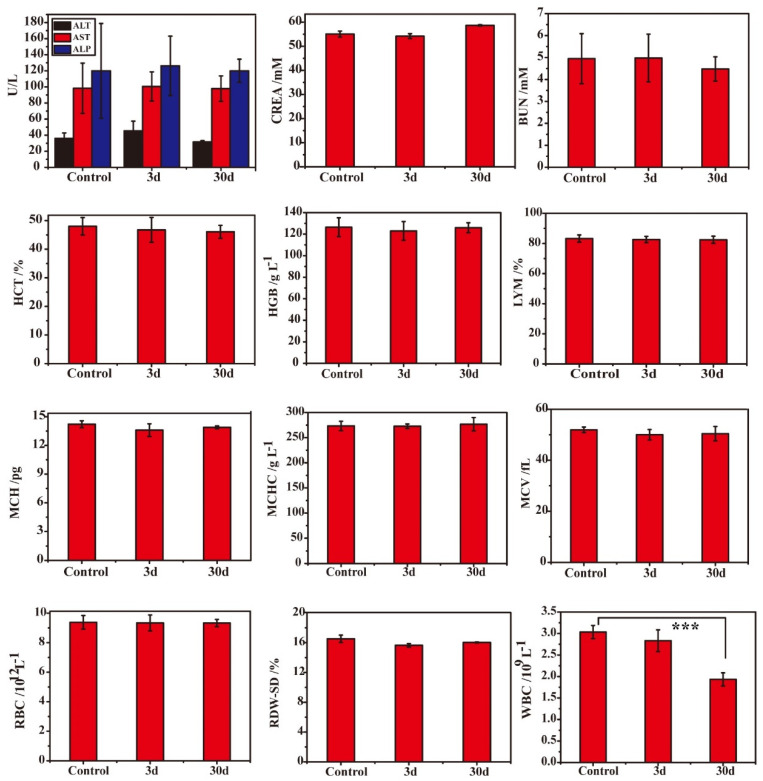
Blood biochemical analysis of Kunming mice after i.v. injection of DMON@GOx/3-MA with a calculated dose of 75 mg/kg at day 3 and day 30. The liver function related alanine aminotransferase (ALT), aspartate aminotransferase (AST), alkaline phosphatase (ALP), and creatinine (CREA), nephric blood urea nitrogen (BUN) and other indices show no significant change after treatment. n = 6, data are the means ± s.d. *** *p* < 0.001, *p* value was analysed by one-way ANOVA.

To further confirm the systemic toxicity of DMON@GOx/3-MA in vivo, Kunming mice intravenously injected with DMON@GOx/3-MA at a calculated dose of 75 mg/kg, were sacrificed at day 3 and day 30. All organ tissues and blood were collected. As shown in Figure 5, no significant changes were detected in all of the blood indices except WBC and no evident physiological and morphological abnormalities in these organs (heart, liver, spleen, lung and kidney) from the mice at day 3 and day 30. Although the number of WBC was significantly decreased after the mice treated with DMON@GOx/3-MA for 30 days, the values are still in the normal range ((0.8–6.8) × 10^9^/L). In addition, histological analyses (hematoxylin-eosin staining, H&E staining) of the organ tissues, such as heart, liver, spleen, lung and spleen of these groups were performed, and there were no evident physiological and morphological abnormalities in these organs (Figure S10). During the 30 days, no significant difference in body weight between the control injection group and the DMON@GOx/3-MA group (Figure S11). All these results indicated that DMON@GOx/3-MA has good biocompatibility and biosafety for tumor therapy.

## 4. Discussion

Starvation therapy is an effective strategy for suppressing tumor growth and tumor survival by blocking blood flow or depleting nutrients/O_2_ supply. Because most of the tumor cells transport and metabolize nutrients at a considerably faster rate than normal cells (due to fast tumor metabolism and proliferation), cancer cells are more sensitive to changes in intracellular glucose and O_2_ concentration [35]. Therefore, compared to normal cells, cancer cells are more sensitive to starvation therapy. GOx effectively depletes glucose and O_2_ in tumor cells and meanwhile produces a considerable amount of H_2_O_2_, which gives GOx a great prospect for application in cancer starvation therapy. However, cancer cells can harness intrinsic protective and adaptative autophagy to eat or digest their own cytoplasmic components for nutritive compensation, limiting the efficiency of starvation therapy. Thus, the combination of tumor starvation therapy with anti-autophagy therapy would deplete exogenous and endogenous nutrition, leading to a remarkable synergetic antitumor outcome. Notably, just as in tumor starvation therapy, tumors cell may also induce adaptative autophagy to counteract other kinds of tumor therapies, such as photothermal therapy, photodynamic therapy, radiotherapy, and part of chemotherapy [11]. Therefore, the anti-adaptative strategy proposed in this study provides a broad-spectrum approach to enhancing tumor therapy.

In this study, we first prepared biodegradable DMONs, and then we aminated DMON using APTES. Through the EDC/NHS chemistry method, we successfully loaded GOx covalently into DMON to prepare DMON@GOx. After that, we successfully loaded the autophagy inhibitor 3-MA into the dendritic pores of DMON to prepare the final formulation-DMON@GOx/3-MA. Through a TGA detection method [36,37], we determined that the loading rates of GOx and 3-MA in DMON@GOx/3-MA were 2.31% and 2.48%, respectively. We found the loading capacities of GOx and 3-MA were relatively fixed. It is difficult for us to adjust the ratio between GOx and 3-MA. We do believe that the ratio between the amount of GOx and 3-MA is relevant to achieve both effects, but other material synthesis methods are needed to address this question. In vitro experiments show that DMON@GOx/3-MA nanoparticles can effectively degrade and release 3-MA in SBF solution. After DMON@GOx/3-MA nanoparticles endocytosed by tumor cells, DMON@GOx/3-MA is transferred to lysosomes and degraded in the acidic physiological conditions of the lysosome. Thus, the dendritic pores will collapse, and the drug will be released. In this process, whether the protein corona is present or not, it does not affect the release of the drug. Moreover, in this study, we use HPLC to detect the release of 3-MA. Therefore, it is difficult for us to evaluate the release of drugs from DMON@GOx/3-MA in a protein environment.

In this study, we found that DMON@GOx exhibited higher cytotoxicity than DMON + GOx groups, and DMON@GOx/3-MA exhibited higher cytotoxicity than DMON + GOx + 3-MA groups, demonstrating the cytotoxicity of nanoparticle formulation is better than the mixture of the components. To determine if autophagy is involved in GOx-mediated cell death, autophagy inhibitor 3-MA was used to perform cell proliferation assay (MTT) and cell apoptotic assay (PI/annexin V and Western blot). All these results demonstrate that 3-MA enhanced the GOx-induced starvation therapy. To further investigate how autophagy is involved in this process, two important proteins LC3B and P62 were used as autophagy markers. LC3BII is a marker of autophagosome formation, which is generated by the conjugation of cytosolic LC3BI to phosphatidylethanolamine (PE) on the surface of nascent autophagosomes. P62 is a marker of autophagic flux, which is an autophagy substrate that is negatively related to autophagy. The reduction of p62 has been regarded as a marker for the increase of autophagic flux [38,39]. Surprisingly, DMON@GOx/3-MA not only induced P62 expression but also induced LC3BII expression, suggesting DMON@GOx/3-MA inhibits autophagy via blocking the autophagy flux but not the formation of autophagosomes. This result is also consistent with the results from immunofluorescence using a tandem fluorescent mRFP-GFP-LC3 (tfLC3) assay. 3-MA inhibited autophagy by inhibiting the formation of autolysosomes but not autophagosomes. This is inconsistent with the reported activity of 3-MA [25,26,27] that inhibits the formation of the autophagosomes. The reason for this interesting phenomenon is unclear. Further studies are required to explore the clear molecular mechanisms of 3-MA activities.

It has been reported that 3-MA has dual functions [40]. Wu et al. reported that 3-MA promoted autophagy under a nutrient-rich condition but inhibited autophagy under a starvation condition. 3-MA can increase the LC3BII/LC3B1 ratio and the expression of P62 to induce autophagy under a nutrient-rich condition. However, such a change of P62 was independent of autophagy and was not the result of autophagy suppression. This phenomenon is similar but different from our results. Although both of our work showed increases of LC3BII/LC3B1 ratio and p62, 3-MA promoted the formation of autolysosome to increase autophagy in Wu’s work [40], while 3-MA inhibited the formation of autolysosome in our work, leading to the blocking of autophagy flux.

In this study, DMON@GOx/3-MA shows relatively modest cytotoxicity in vitro. However, the cytotoxicity of DMON@GOx/3-MA in vivo is relatively outstanding. We hypothesize that the levels of glucose/O_2_ are higher in an in vitro culture system than in vivo. There is 4.5 g/L of glucose (25 mM) in a DMEM culture medium, and an adequate concentration of O_2_ in incubators, which are more than available in physiological conditions (approximately 7 mM glucose and an appropriate amount of O_2_). This phenomenon is also consistent with the studies published before [16]. In additionally, as shown in Figure 4 and other group’s studies [41], the 4T1 tumor appears necrosis after transplantation for 15 days, which indicates that the tumors were short of nutrients (glucose or O_2_) in vivo, resulting in the higher sensitivity to tumor starvation therapy. Therefore, the starvation therapy efficiency of DMON@GOx/3-MA in vivo is better than in vitro. It has been shown that there is no significant difference in the tumor volume after administration of DMON@GOx/3-MA (i.t. or i.v.) in Figure 4B. The reason is that we performed the i.v. administration three times on day 1, day 2, and day 3 to suppress the tumor growth, while performed i.t. administration only once. It is interesting to note that DMON@GOx/3-MA i.v. for three consecutive days can effectively inhibit tumor growth, which further demonstrated the effective cancer treatment of DMON@GOx/3-MA.

## 5. Conclusions

In summary, an enhanced tumor starvation therapy was achieved via autophagy inhibition with the GOx and 3-MA co-loaded DMON. After DMON@GOx/3-MA accumulates in tumors, GOx locally consumes glucose and oxygen to starve cells, leading to tumor-associated metabolic abnormalities that activate autophagy. Furthermore, 3-MA severely blocks auto-phagolysosome formation, augmenting the efficacy of tumor starvation. Our study offers an anti-cell adaptation strategy for enhanced tumor starvation therapy via autophagy regulation in cancer cells, which is promising for effective cancer treatment. This anti-cell adaptative strategy would also be beneficial to other autophagy-related cancer therapies such as oxidative stress therapy, hyperthermia therapy, etc.

## Data Availability

The data presented in this study are available in the article and its Supplementary Materials.

## Supplementary Materials

The following are available online at https://www.mdpi.com/article/10.3390/biom11091363/s1, Figure S1: XRD pattern of DMON nanoparticles, Figure S2: DLS data of DMON@GOx/3-MA nanoparticles in different solutions, Figure S3: FTIR spectra of GOx, 3-MA, DMON-NH_2_ and DMON@GOx/3-MA, Figure S4: TG curves of GOx and 3-MA, Figure S5: Standard curve of H_2_O_2_ detected by titanous sulfate reagent, Figure S6: Confocal laser scanning microscopy (CLSM) images of FITC-labeled DMON@GOx/3-MA, Figure S7: Cell viability of 4T1 cells treated with different concentrations of 3-MA, Figure S8: Bio-TEM images of 4T1 cells treated with DMON@GOx and DMON@GOx/3-MA, Figure S9: Digital photos of 4T1 tumor-bearing mice after different treatments, Figure S10: H&E-stained images of tissue sections, Figure S11: Time-dependent body weight record of Kunming mice.
