# Supplementary material for: Enhanced Cancer Starvation Therapy Based on Glucose Oxidase/3-Methyladenine-Loaded Dendritic Mesoporous OrganoSilicon Nanoparticles
Source: Biomolecules. 2021 Sep 14;11(9):1363. doi: 10.3390/biom11091363 (PMC8468959; doi:10.3390/biom11091363)
Supplement: Supplementary file 1 [file biomolecules-11-01363-s001.zip › biomolecules-1271039-supplementary.pdf]

Supplementary Materials

Enhanced Cancer Starvation Therapy Based on Glucose Oxidase/3-Methyladenine-Loaded Dendritic Mesoporous Organosilicon Nanoparticles

Fan Wu [1,†], Yang Liu [2,†], Hui Cheng [2,†], Yun Meng [1], Jieyun Shi [1], Yang Chen [1] and Yelin Wu [1,*]

[1] Tongji University Cancer Center, Shanghai Tenth People's Hospital, Tongji University School of Medicine, Shanghai 200072, China; 18844191898@163.com (F.W.); 18356070780@163.com (Y.M.); 15800870206@163.com (J.S.); fc9091@163.com (Y.C.)

[2] Shanghai Key Laboratory of Green Chemistry and Chemical Processes, School of Chemistry and Molecular Engineering, East China Normal University, Shanghai 200062, China; ly92_pharmacy@163.com (Y.L.); haotianchenghui@163.com (H.C.)

* Correspondence: sk_wuyelin@tongji.edu.cn

† These authors contributed equally to this work.

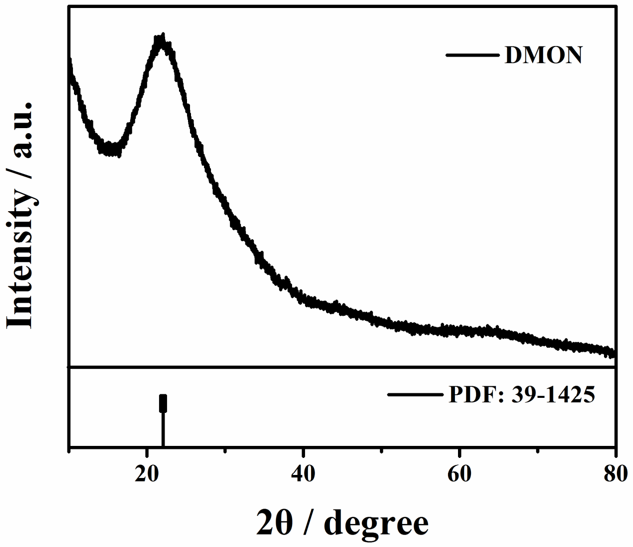

Figure S1. X-ray diffraction (XRD) pattern of DMON nanoparticles.

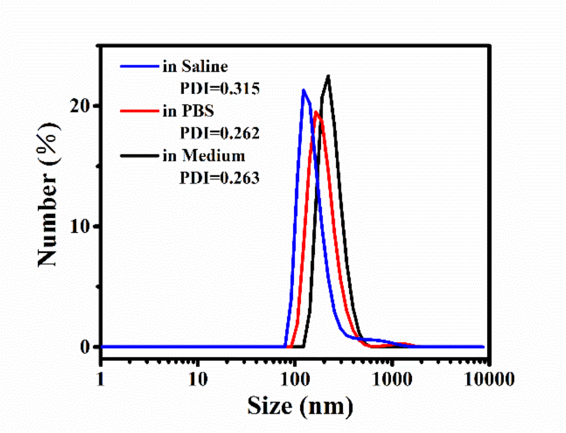

Figure S2. Dynamic light scattering (DLS) data of DMON@GOx/3-MA nanoparticles in different solutions.

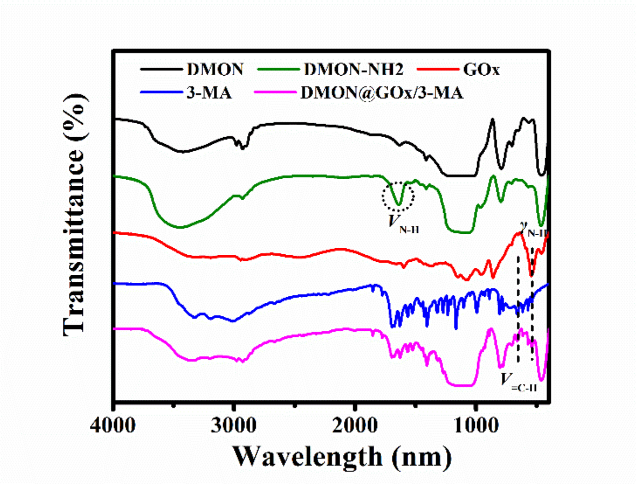

Figure S3. Fourier transform infrared (FTIR) spectra of GOx, 3-MA, DMON, DMON-NH_2 and DMON@GOx/3-MA.

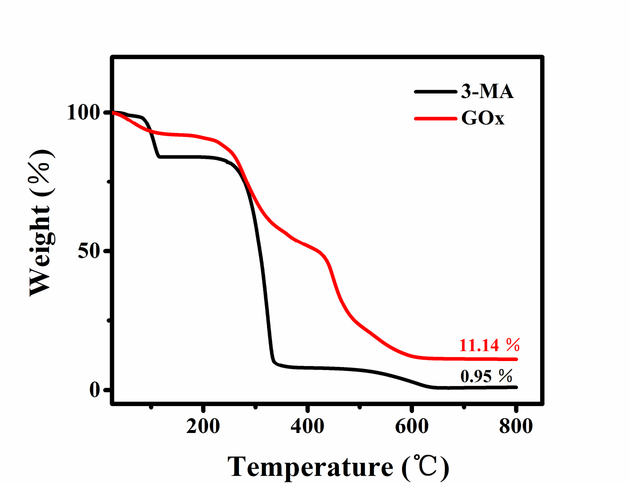

Figure S4. Thermogravimetric (TG) curves of GOx and 3-MA.

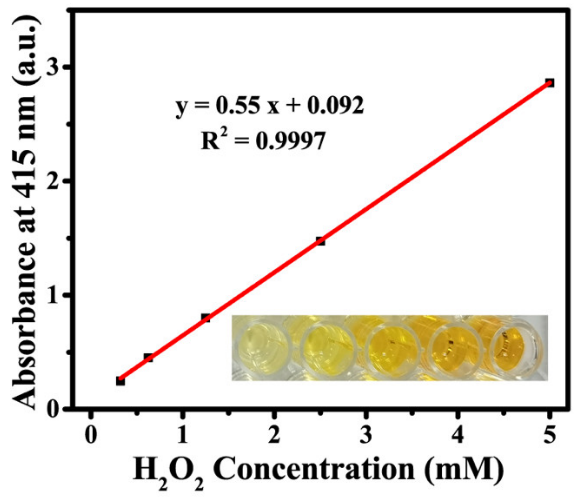

Figure S5. Standard curve of H_2O_2 detected by titanous sulfate reagent.

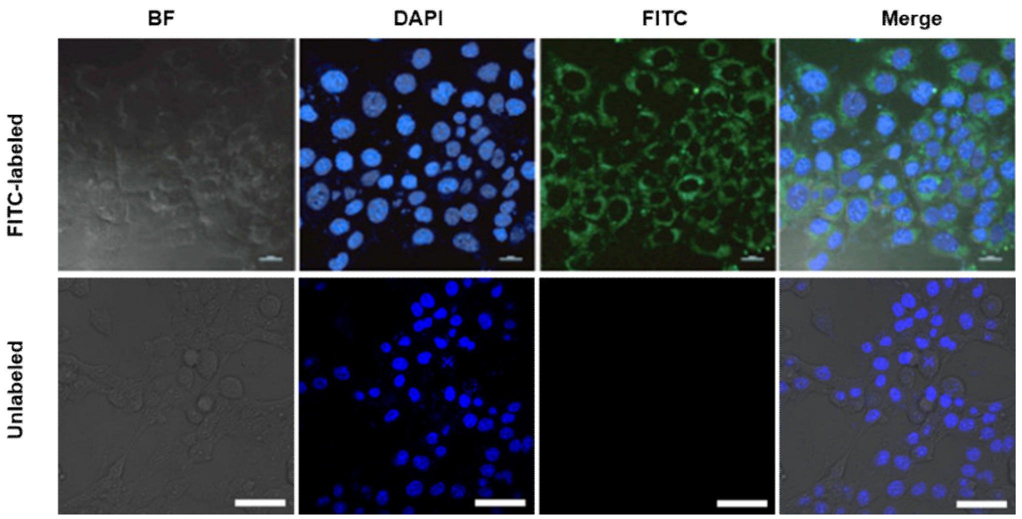

Figure S6. Confocal laser scanning microscopy (CLSM) images of FITC-labeled and unlabeled DMON@GOx/3-MA in the 4T1 cells (scale bar = 50 μm).

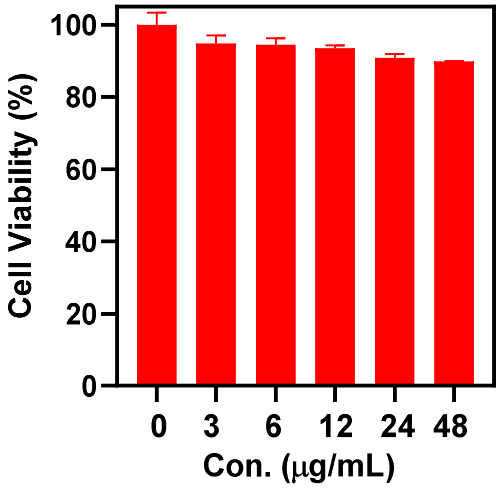

Figure S7. Cell viability of 4T1 cells treated with different concentrations of 3-MA for 24 h

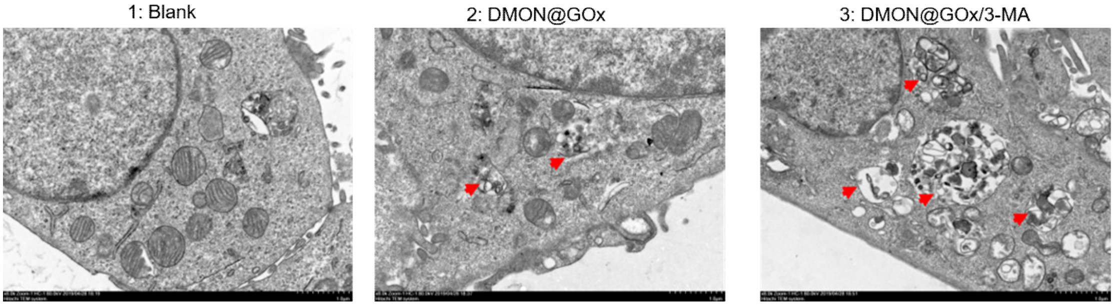

Figure S8. Bio-TEM images of 4T1 cells treated with DMON@GOx and DMON@GOx/3-MA for 24 h.

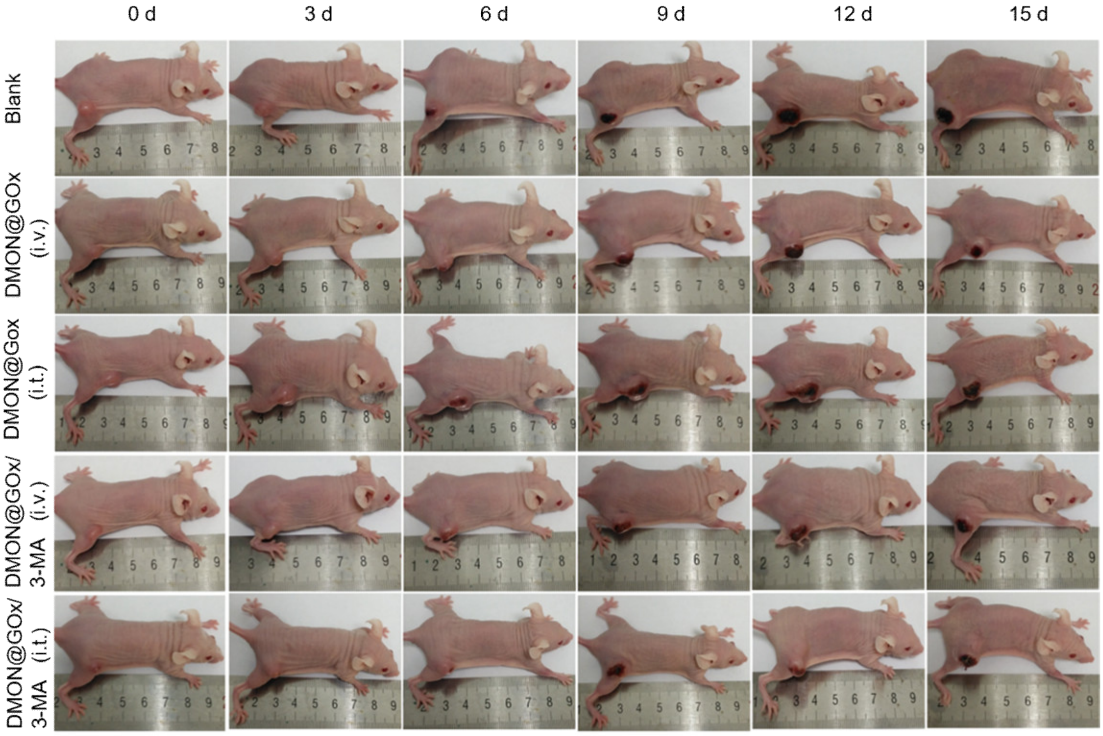

Figure S9. Digital photos of 4T1 tumor-bearing mice after different treatments.

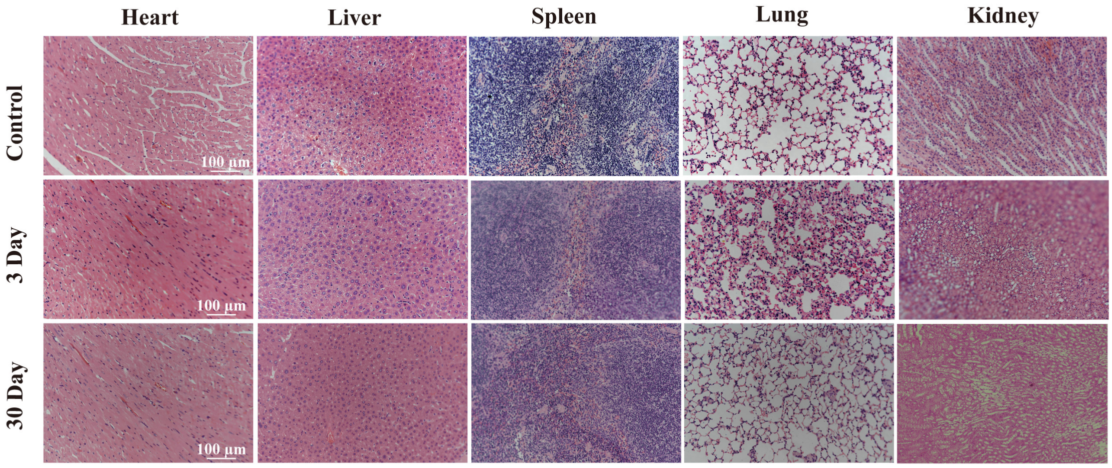

Figure S10. Pathological H&E-stained images of tissue sections from heart, liver, spleen, lung and kidney of the mice treated with DMON@GOx/3-MA. The tissue sections were harvested in 3 and 30 days after the intravenous injection of a 50 mg/kg dosage, showing no significant change of physiological and morphological abnormalities (Scale bars, 100 μm).

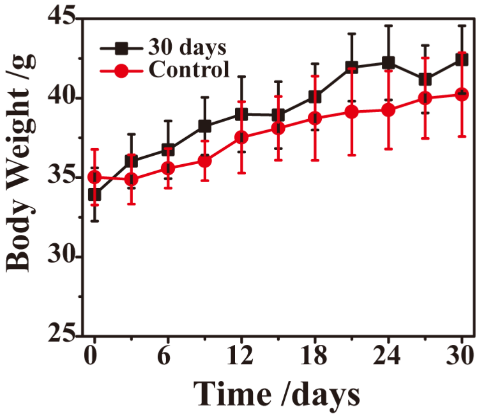

Figure S11. Time-dependent body weight record of Kunming mice after i.v. injection of DMON@GOx/3-MA with a calculated dose of 75 mg/kg (n = 6, mean ± s.d.).
